# Lactylation and macrophage polarization from a clinical perspective: pathological mechanisms and therapeutic potential

**DOI:** 10.3389/fimmu.2026.1840915

**Published:** 2026-07-01

**Authors:** Chenfei Ma, Jinyan Han, Yu Tian

**Affiliations:** Department of General Surgery, Shengjing Hospital of China Medical University, Shenyang, Liaoning, China

**Keywords:** lactylation, macrophage polarization, pathological mechanism, post-translational modification, therapeutic strategy

## Abstract

As an emerging epigenetic modification, lactylation has attracted increasing attention in studies of macrophage activation in recent years. Accumulating evidence indicates that lactylation not only regulates macrophage gene-expression programs and polarization trajectories, but also contributes to the pathogenesis and prognosis of a wide range of diseases. In this review, we summarize fundamental concepts related to lactate metabolism and protein lactylation, and we discuss how lactylation shapes macrophage function and plasticity, with particular emphasis on its mechanistic roles under distinct pathophysiological conditions. In addition, we examine the regulatory networks through which lactylation modulates macrophage polarization and highlight its potential translational applications in clinical intervention. Collectively, this review aims to provide updated insights and therapeutic perspectives to support disease prevention, delay progression, and improve patient outcomes. We also emphasize the limitations of causal inference in current studies, contextualize the classical M1/M2 nomenclature, and synthesize how lactate flux, microenvironmental cues, thresholds of lactylation, and writer/eraser/reader balance may determine divergent disease outcomes.

## 1 Introduction

In 2019, the discovery of histone lactylation revealed a previously unrecognized class of protein post-translational modifications (PTMs). Mediated by lactyl–coenzyme A, a metabolite derived from lactate, histone lactylation contributes to immune-cell functional reprogramming through mechanisms including the regulation of gene expression (1). With the rapid expansion of research in this field, lactylation has also been identified on a wide range of non-histone proteins (2, 3). Lactate was once regarded merely as a byproduct of anaerobic glycolysis; however, subsequent studies have shown that glucose metabolism can generate lactate through glycolysis even under oxygen-replete conditions, thereby providing rapid energy support for both normal and malignant cells (4, 5). Moreover, lactate accumulation not only amplifies inflammatory responses but also promotes the establishment of an immunosuppressive microenvironment (6–8), thereby facilitating tumor immune evasion.

Notably, lactate and lactylation modification have emerged as vital metabolic and epigenetic modulators of immune function. As pivotal innate immune cells with diverse regulatory functions, macrophages are highly responsive to lactate-induced molecular and phenotypic alterations (9), making them a key research object in lactylation-related immune regulation. In this review, the terms M1-like and M2-like are used as heuristic descriptors of activation programs, rather than as fixed lineages or mutually exclusive *in vivo* identities. The canonical M1/M2 framework was mainly developed from classical *in vitro* stimulation systems, such as IFN-γ/LPS-induced inflammatory macrophages and IL-4/IL-13-induced reparative macrophages, whereas macrophages in diseased tissues often occupy continuous, mixed, or disease-specific states (10–15). For example, tumor-associated macrophages (TAMs) and disease-associated macrophages (DAMs) may co-express inflammatory, reparative, angiogenic, metabolic, and immunosuppressive modules and therefore cannot be rigidly assigned to a binary M1 or M2 category. Accordingly, throughout this review we use M1-like and M2-like terminology only when it helps organize the literature, while emphasizing macrophage heterogeneity, tissue context, and functional plasticity.

Therefore, elucidating the molecular mechanisms by which lactylation modulates macrophage polarization will not only refine current frameworks of immune regulation but also reveal new targets for immunotherapy in relevant diseases. In this review, we summarize the roles and molecular mechanisms of lactylation in the regulation of macrophage polarization and discuss how lactylation-driven changes in macrophage functional states influence disease initiation and progression across a spectrum of pathophysiological processes, including tissue injury and repair, infectious and inflammatory diseases, cardiovascular and metabolic disorders, cancer, fibrosis, and central nervous system degenerative diseases. Finally, we highlight emerging therapeutic strategies targeting macrophage lactylation and outline key challenges and future directions in this rapidly evolving field.

## 2 Overview of lactate metabolism and lactylation modification

### 2.1 Lactate metabolism

**Figure 1:**
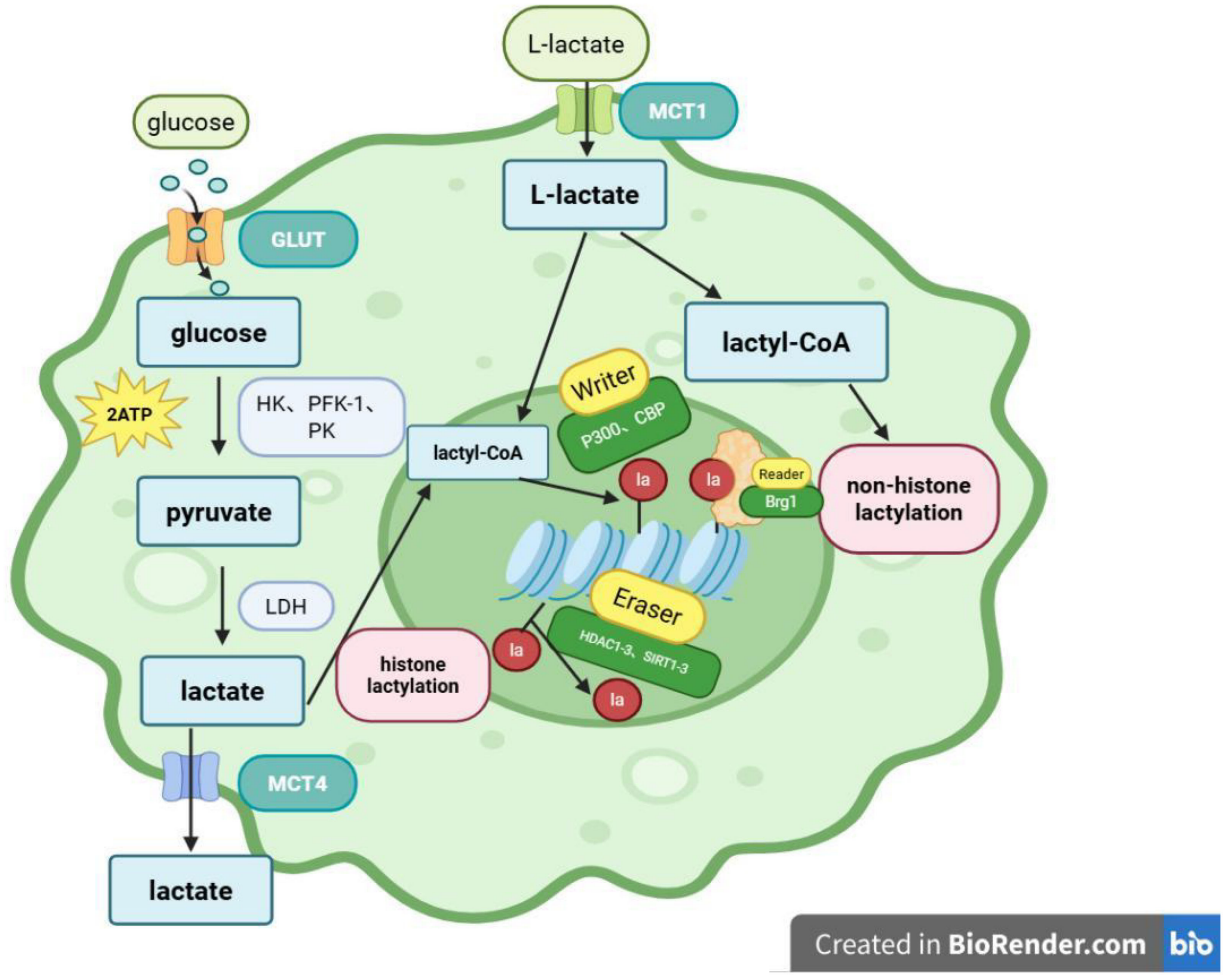
Lactate metabolism and histone lactylation. Lactate generated through glycolysis is transported between cells via monocarboxylate transporters MCT1 and MCT4. Acting as a metabolic substrate, lactate supports lactylation and thereby regulates gene expression through epigenetic mechanisms. The histone lactylation process is illustrated as follows: L-lactate derived from multiple metabolic pathways combines with intracellular coenzyme A to form lactyl-CoA. Lactyl-CoA subsequently enters the nucleus, where its lactyl group is conjugated to histone lysine residues by writers. Readers recognize and interpret the lactylation mark and relay the signal downstream to modulate transcription of related genes. Finally, erasers remove the lactyl group from lysine residues, allowing these sites to participate in other histone post-translational modifications.

Glucose is the primary energy source for the human body. Under aerobic conditions, glucose is fully oxidized through glycolysis, the tricarboxylic acid (TCA) cycle, and oxidative phosphorylation, ultimately producing carbon dioxide (CO_2_). By contrast, under tissue hypoxia, glucose is converted to lactate via anaerobic glycolysis. Although this pathway yields only two molecules of ATP per molecule of glucose and is therefore energetically inefficient, it provides ATP rapidly; this process is commonly referred to as lactic acid fermentation. Nearly a century ago, Warburg observed that cancer cells preferentially convert large amounts of glucose to lactate even in the presence of sufficient oxygen, thereby supporting rapid growth, proliferation, and metastasis. This metabolic phenotype is known as the Warburg effect (5). Subsequent studies have further shown that this lactate-associated mode of rapid energy production is not restricted to cancer cells but is also widely adopted by normal cells, including activated T cells, B cells, macrophages, and intestinal epithelial cells (16). Because glycolysis-derived lactate can be converted to lactyl-CoA and subsequently used for histone and non-histone lactylation, the overall metabolic route from glucose to lactate, lactate shuttling, and lactylation is summarized schematically in **Figure 1**.

### 2.2 Lactate shuttle

The lactate shuttle theory was proposed by George Brooks and colleagues (17). This theory posits that, once generated intracellularly, lactate not only exerts effects within the producing cell but can also function as an energy carrier and signaling molecule that is transported among different cells, tissues, and organs. The lactate shuttle contributes to the maintenance of acid–base balance and metabolic homeostasis and is involved in a broad range of biological processes, including energy regulation, immune tolerance, memory formation, wound healing, and cancer growth and metastasis (18).

Monocarboxylate transporters (MCTs) are key mediators of the lactate shuttle. They facilitate bidirectional lactate transport across the plasma membrane via a 1:1 symport mechanism that couples lactate flux to proton (H^+^) movement (19). To date, 14 members of the MCT family (MCT1–MCT14) have been identified, among which MCT1 and MCT4 are the most extensively studied with respect to lactate transport (20). MCT1 exhibits a high affinity for lactate and primarily mediates lactate uptake, whereas MCT4 has a lower affinity but a higher transport capacity and is mainly responsible for lactate export from highly glycolytic cells, such as tumor cells and macrophages (21, 22). In addition, the concentration gradients of lactate and protons partially determine the directionality of MCT-mediated transport (23). Notably, MCT4 deficiency enhances H3K18 lactylation at the loci of reparative genes, including *IL-10* and *PDHA1*, thereby promoting transcription of the *MCT4* gene (24). Importantly, MCT-mediated lactate transport is bidirectional and depends on the extracellular-to-intracellular lactate gradient, proton gradient, and isoform expression. Thus, MCT4 inhibition can increase intracellular lactate retention and lactylation in macrophages, whereas high MCT4 expression in highly glycolytic cells can also raise extracellular lactate and indirectly enhance lactylation in neighboring lactate-importing cells. These apparently divergent observations therefore reflect different compartments of lactate flux rather than a simple one-directional relationship between MCT4 abundance and lactylation.

### 2.3 Lactylation modification in epigenetic modifications

Epigenetic modifications refer to chemical changes in molecules such as DNA, histones, and non-coding RNAs (e.g., miRNAs, lncRNAs, and circRNAs) that regulate gene expression without altering the underlying DNA sequence. These modifications play essential roles in cell differentiation, organismal development, and disease onset and progression. In recent years, histone post-translational modifications (PTMs) have attracted substantial attention. Histones are highly conserved basic proteins that constitute the fundamental structural framework of eukaryotic chromatin. They are generally classified into linker histones (H1) and core histones (H2A, H2B, H3, and H4). Histone PTMs include methylation, acetylation, phosphorylation, ubiquitination, SUMOylation, and ADP-ribosylation (25). Collectively, these modifications regulate transcriptional programs, participate in DNA damage repair, and influence DNA replication. In addition, they can serve as “docking sites” for effector proteins, thereby contributing to the maintenance of cellular homeostasis and function.

#### 2.3.1 Histone lactylation and non-histone lactylation

In 2019, the research team led by Yingming Zhao at the University of Chicago first confirmed lysine lactylation as an endogenous protein post-translational modification *in vivo*. By developing a pan–anti-lysine lactylation antibody, they identified histone lysine lactylation (Kla) and mapped 26 and 16 histone Kla sites in MCF-7 cells and mouse bone marrow–derived macrophages (BMDMs), respectively (1). Current evidence suggests that lactate, as an α-hydroxycarboxylic acid, exists as two stereoisomers—L-lactate and D-lactate—yet histone lactylation predominantly involves L-lactate (26). This selectivity is thought to be largely attributable to the low physiological abundance of D-lactate, rather than to stereochemical constraints per se.

Beyond histones, widespread lactylation has also been detected on non-histone proteins across multiple subcellular compartments, including the nucleus, mitochondria, endoplasmic reticulum, and plasma membrane (27). For example, in hepatitis B virus–associated hepatocellular carcinoma, proteomic profiling identified 9,275 lysine lactylation sites, of which 9,256 were mapped to non-histone proteins. In addition, a proteomic study of the fungal pathogen responsible for Botrytis cinerea also reported 273 lysine lactylation sites (28). Mechanistically, lactate has been shown to directly promote lactylation of the nuclear protein HMGB1 in macrophages during sepsis (2). Moreover, lactylation of *PKM2* at K62 contributes to inflammatory adaptation in M1 macrophages (29), whereas lactylation of *SQSTM1* at K63 serves as a key event that activates the *STAT3/CCL2* signaling pathway (30). Similar to histone lactylation, non-histone lactylation is implicated in diverse cellular processes, including gene transcription, DNA damage repair, cell division, signal transduction, protein folding, autophagy, and metabolism (31).

#### 2.3.2 Formation mechanism of lactylation modification

Briefly, lactylation involves the covalent attachment of a lactyl group derived from lactate to a lysine residue on a target protein via the ϵ-amino group. In principle, elevated lactate concentrations could enable direct, non-enzymatic lactylation of lysine residues. However, accumulating evidence suggests that most lactylation events are catalyzed enzymatically, using the high-energy intermediate lactyl-CoA as the acyl donor (32). This enzymatic lysine lactylation is governed by three functional classes of regulatory proteins—”writers”, “erasers”, and “readers”. As the terms imply, writers transfer lactyl groups from lactyl-CoA to lysine residues on substrate proteins; erasers remove the modification, thereby maintaining the dynamic equilibrium of lactylation; and readers comprise domains that selectively recognize and bind lactylation marks, facilitating interpretation of lactylation signals and downstream signal propagation (**Figure 1**).

##### 2.3.2.1 Writer

During lactylation, writers largely determine the efficiency and extent of lactyl mark installation. Among the best-characterized lactyltransferases are p300 and CREB-binding protein (CBP), both members of the histone acetyltransferase (HAT) family. Zhang et al. demonstrated a key role for p300 in histone lactylation by overexpressing *p300* in HEK293T cells and knocking down *p300* in HCT116 and HEK293T cells, followed by quantification of lactylation levels under each condition (1). Consistently, pharmacological inhibition of p300/CBP using C646 markedly reduced lactylation levels in macrophages. In addition, lysine acetyltransferases such as HBO1 and KAT8 have also been reported to function as lactyltransferases and to catalyze lactylation. HBO1 preferentially promotes lactylation at histone H3K9 (33), whereas KAT8 can lactylate multiple oncoproteins (e.g., eEF1A2) and enhances colorectal cancer cell proliferation within a high-lactate tumor microenvironment (34). Accordingly, targeting *KAT8* to suppress oncoprotein lactylation may represent a promising therapeutic strategy for colorectal cancer.

##### 2.3.2.2 Eraser

The currently identified lactylation erasers are mainly histone deacetylases (HDACs) 1–3 and sirtuins (SIRTs) 1–3. *In vitro* screening assays using synthetic peptides and core histones as substrates have been conducted to evaluate delactylase activity, revealing that the nuclear deacetylases HDAC1–3 exhibit the highest delactylation efficiency. Among them, HDAC1 and HDAC3 markedly reduce lactylation at H4K5 (H4K5la) (26). In addition, SIRT1, SIRT2, SIRT3, and SIRT5 are all capable of removing lactyl groups from lysine residues, with SIRT2 displaying higher catalytic activity than the other family members. Notably, the amino acid context surrounding the lactylated lysine affects SIRT2-mediated delactylation; specifically, delactylation efficiency is substantially reduced at sites adjacent to glycine or proline residues (35). In neuroblastoma, *SIRT2* knockout significantly increases lactylation at H3K18, H4K8, and H4K12, thereby promoting tumor cell proliferation and metastasis (36). Accordingly, strategies that enhance *SIRT2* expression or activity may represent a potential therapeutic avenue for neuroblastoma.

##### 2.3.2.3 Reader

During pluripotent stem cell reprogramming, Brg1 has been reported to function as a reader that specifically binds H3K18la and promotes its enrichment at the promoters of reprogramming-associated genes, thereby facilitating the reprogramming process (37). Although the repertoire of lactylation readers remains incompletely characterized, future studies should investigate whether established readers of other lysine acylation marks—such as YEATS domain–containing proteins, bromodomain-containing proteins, and PHD zinc finger proteins—can also recognize lactylation, potentially through cross-talk or cross-recognition mechanisms (**Table 1**).

**Table 1:** Overview of lactylation writers, erasers, and readers.

Category	Representative molecule	Main target(s)	Evidence/approach	Context and references
Writer	p300/CBP	Histone Kla, including macrophage H3/H4 sites	Gain/loss of function; C646; LC-MS/MS; ChIP assays	Macrophages and tumor models (1)
Writer	HBO1/KAT7	H3K9la and transcriptional regulation	Enzymatic assays; chromatin profiling; genetic perturbation	Chromatin regulation (33)
Writer	KAT8/MOF	Non-histone oncoproteins, e.g., eEF1A2 K408	Proteomics; mutagenesis; rescue experiments	High-lactate colorectal cancer context (34)
Eraser	HDAC1/2/3	Histone Kla, including H4K5la	In vitro delactylase assays; HDAC inhibition	Class I HDAC-sensitive delactylation (26)
Eraser	SIRT1/2/3/5	Histone and non-histone Kla; SIRT2-sensitive H3K18la/H4K8la/H4K12la	Knockout/overexpression; enzymatic assays; LC-MS/MS	Neuroblastoma and broader nuclear/mitochondrial contexts (35, 36)
Reader	Brg1; candidate YEATS/bromodomain/PHD proteins	H3K18la-enriched promoters; potential acyl-lysine cross-recognition	ChIP/binding assays; structural and peptide-pulldown studies needed	Defined in reprogramming; macrophage readers remain incompletely mapped (37)

## 3 Macrophage polarization

**Figure 2:**
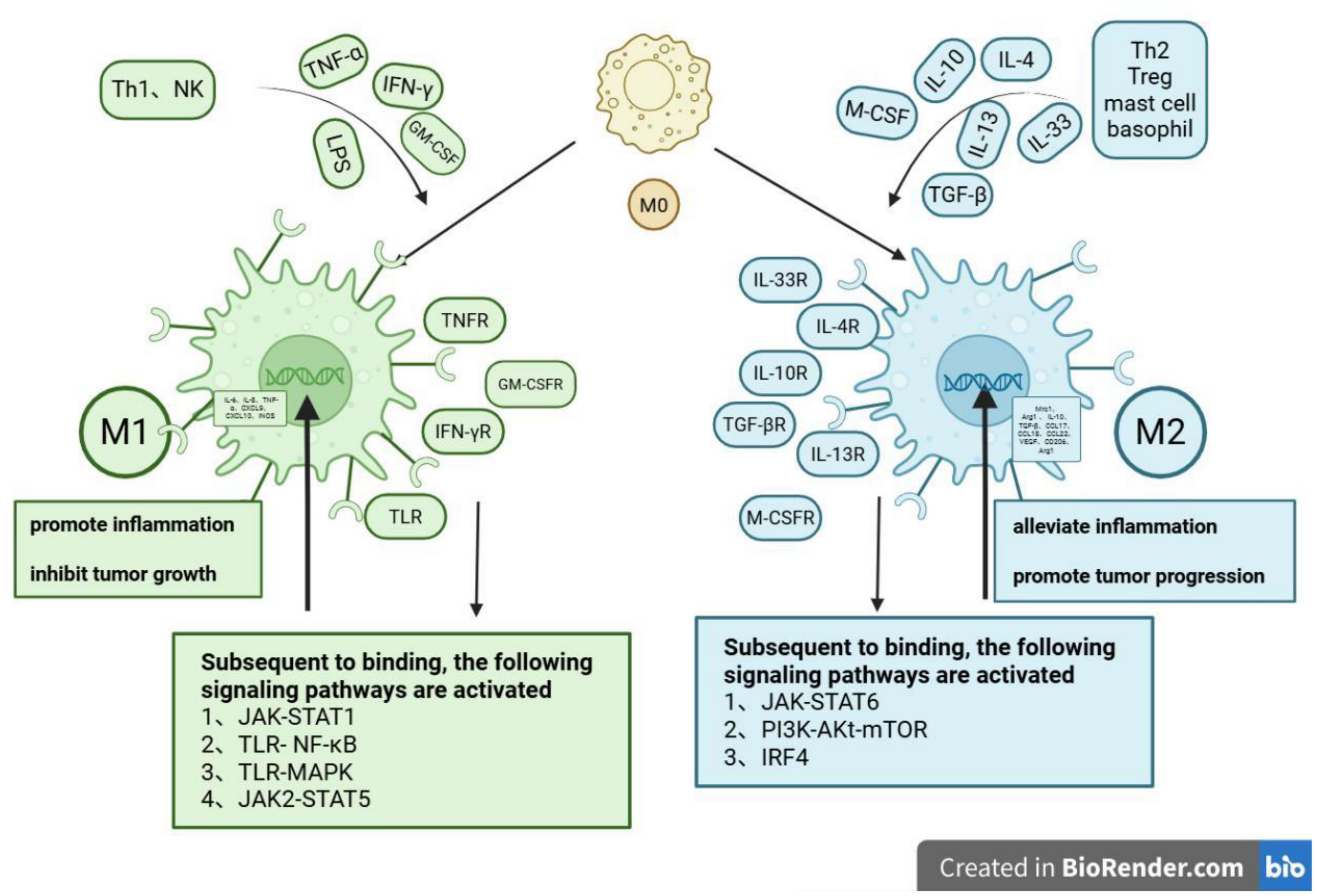
Mechanisms of macrophage polarization Naïve M0 macrophages can polarize into either M1 or M2 subsets. The M1 phenotype is induced by stimuli such as IFN-γ, GM-CSF, TNF-α, and LPS and is characterized by pro-inflammatory and anti-tumor functions. In contrast, the M2 phenotype is driven by cytokines including IL-4, IL-10, IL-13, IL-33, TGF-β, and M-CSF and is associated with anti-inflammatory and pro-tumor activities. These two subsets express distinct surface receptors that sense specific inducers and activate corresponding signaling pathways. Ultimately, these pathways promote the expression of subset-specific genes and distinct cytokine profiles, leading to divergent biological outcomes.

Macrophages, key innate immune cells, are essential components of the immune system and also contribute critically to adaptive immune responses. Although circulating monocytes are continuously generated from bone marrow hematopoietic stem cells and can differentiate into macrophages after entering tissues, this is not the only developmental route. Many tissue-resident macrophages are established during embryonic hematopoiesis, including yolk-sac- and fetal-liver-derived waves, and can self-maintain locally with limited replacement by adult monocytes under steady-state conditions (13, 38, 39). These ontogenic differences are biologically relevant because microglia, Kupffer cells, alveolar macrophages, intestinal macrophages, and monocyte-derived inflammatory macrophages can differ substantially in enhancer landscapes, metabolic dependencies, and responsiveness to lactate/lactylation cues. Their principal functions include phagocytosis of foreign materials, antigen presentation, tissue remodeling, and regulation of immune responses (**Figure 2**).

Apart from ontogenetic variations, macrophages display profound functional plasticity, allowing them to dynamically alter their activation profiles in response to changing tissue microenvironments (40). In response to cues from distinct microenvironments, macrophages can adopt activation programs that are often described as M1-like or M2-like, but these terms should be understood as endpoints of a broad activation spectrum rather than mutually exclusive states (12–15). The M2 subclassification into M2a, M2b, M2c, and M2d is useful for summarizing selected *in vitro* and experimental observations, but it does not fully capture macrophage diversity in tumors, neurodegenerative disease, metabolic inflammation, or tissue repair. For instance, TAMs may simultaneously express angiogenic, scavenger, inflammatory, and immunosuppressive genes, while DAMs in neurodegenerative diseases can acquire lipid-handling and phagocytic programs that do not map cleanly onto M1/M2 terminology. During macrophage activation, cytokines and damage-associated or pathogen-associated signals bind to specific receptors and activate downstream transcription factors, thereby inducing context-specific gene programs through multiple signaling cascades. For example, IFN-γ, TNF-α, and LPS activate pathways involving NF-κB, STAT1, and IRF5, whereas IL-4, IL-10, and TGF-β can engage STAT3/STAT6, IRF4, and PPARγ-related programs (41). These pathways—including JAK–STAT, NF-κB, and MAPK signaling—collectively shape macrophage functional states and their roles in immune responses (42–47).

Macrophages actively remodel metabolic pathways in response to microenvironmental changes, a process referred to as macrophage metabolic reprogramming (48). Macrophage activation and functional heterogeneity are tightly linked to metabolic reprogramming, which serves as a central determinant governing macrophage phenotypic switching and functional specification. In classical mouse *in vitro* systems, inflammatory M1-like macrophages are often characterized by enhanced glycolysis and disrupted TCA-cycle activity, whereas IL-4/IL-13-stimulated M2-like macrophages frequently rely more on mitochondrial oxidative metabolism and fatty-acid utilization (49–51). However, these patterns should be interpreted as model-dependent tendencies rather than universal rules. *In vivo* macrophages may combine glycolysis, oxidative phosphorylation, lipid uptake, and amino-acid metabolism according to tissue niche, ontogeny, oxygen availability, and disease stage. Arginine metabolism provides one example: mouse inflammatory macrophages can use inducible nitric oxide synthase to generate nitric oxide, whereas repair-associated programs often involve arginase-dependent ornithine and polyamine production; in human macrophages, these markers are more variable and should not be used alone to infer functional identity (11, 51).

A further limitation is that many metabolic signatures commonly assigned to M1-like or M2-like macrophages are derived from mouse BMDM or peritoneal macrophage systems. Markers such as inducible nitric oxide synthase activity, arginase 1 expression, and strict glycolysis-versus-oxidative-phosphorylation separation are less robust and less universal in human macrophages (12, 14, 15).

## 4 The effect of lactylation modification on macrophage polarization in pathophysiological processes

### 4.1 Tissue injury and repair

During post-injury tissue repair, M2-type macrophages play a pivotal role. By secreting anti-inflammatory mediators such as IL-10 and TGF-β, they suppress the production of pro-inflammatory cytokines, including TNF-α, IL-1β, and IL-6, thereby dampening the activity of M1-type macrophages and other pro-inflammatory cells and promoting resolution of inflammation. In addition, M2-type macrophages release a range of growth factors—such as TGF-β, vascular endothelial growth factor (VEGF), platelet-derived growth factor (PDGF), and insulin-like growth factor (IGF)—that support cell proliferation, migration, and differentiation, as well as neovascularization. Accordingly, the phenotypic transition of macrophages from an M1-like to an M2-like state is a key determinant of tissue repair outcomes. **Table 2** summarizes the effects of lactylation on macrophage biology and outlines the associated pathological mechanisms across related diseases.

**Table 2:** Effects of lactylation on macrophage-lineage cells and associated pathological mechanisms in related diseases.

Disease/process	Lactylation pattern	Macrophage-lineage outcome	Main evidence/mechanism	Key references
Tissue injury and repair	Late glycolysis-derived histone Kla increases repair genes	Shift toward reparative/M2-like programs	H3K18la/H4K12la promotes Il10, Arg1, Lrg1, Vegf-a or Spp1-related repair modules	(51–58)
Acute infection and sepsis	Lactate-Kla can be protective or harmful depending on substrate	Resolution-like macrophage programs or endothelial injury	MSM/type I IFN axis regulates H3K18la; HMGB1/CIRP lactylation contributes to sepsis injury	(2, 59–63)
Chronic inflammation/barrier disease	TLR-linked glycolysis and LDH-dependent lactate affect histone Kla	Repair, mucosal healing, or suppressed M1-like inflammation	BCAP/TAK-242/GQD act at different nodes of the TLR-glycolysis-lactylation axis	(64–68)
Atherosclerosis/vascular inflammation	MCT4-dependent lactate flux and MeCP2/Sox10 Kla	Protective reparative macrophages or detrimental VSMC transdifferentiation	Cell compartment and lactate-gradient direction explain divergent effects	(3, 69, 70)
Metabolic cardiomyopathy/metabolic disease	FFA- or G6PT-linked lactate accumulation induces H4K12la/H3K18la	M1-like inflammation or inflammasome restraint	HIF-1α and ALKBH5/m6A/NLRP3 pathways connect lactylation to metabolic inflammation	(71–73)
Tumor microenvironment	Tumor-derived lactate raises histone/non-histone Kla	TAM immunosuppression, M2-like programs, immune escape	H3K18la, ENSA K63la, PRKN, TNFSF9 and glycolysis-Kla circuits support TAM-driven progression	(30, 74–78)
Fibrosis-associated disease	Glycolysis-induced Kla activates pro-fibrotic genes	Profibrotic macrophage programs	PM2.5/silica and fibroblast lactate increase TGF-β, VEGF, PDGF, Thbs1 and Opn-associated pathways	(79–83)
CNS degenerative disease	Microglial H4K12la/H3K9la increases in AD/PD models	Pro-inflammatory microglial activation	Glycolysis-H4K12la-PKM2 and H3K9la-SLC7A11 axes sustain neuroinflammation	(73, 84, 85)

#### 4.1.1 Wound healing and tissue repair

In the early phase of tissue injury, macrophages typically adopt an M1-like phenotype to meet the high energetic demands required for rapid responses to pathogen invasion and tissue damage. Accordingly, M1 macrophages generate abundant lactate via aerobic glycolysis, and lactate production promotes histone lactylation. Notably, lactylation accumulates at promoter regions of M2-associated genes during the late stage of M1 macrophage activation (52). This accumulation enhances transcription of M2-related genes and facilitates the expansion of M2 macrophages, thereby restraining excessive inflammation, promoting fibroblast proliferation and collagen deposition, stimulating neovascularization, and creating a permissive microenvironment for tissue remodeling (51, 53, 54).Further studies indicate that triggering receptor expressed on myeloid cells 2 (TREM2) plays a critical role in macrophage transition toward a reparative phenotype. Using TREM2-knockout mice, investigators have shown that lactylation alleviates impaired angiogenesis and delayed wound healing in injured skin by stabilizing hypoxia-inducible factor-1α (HIF-1α) protein expression (55). Collectively, these findings suggest that lactylation may function as a molecular “switch” that drives macrophage polarization from a pro-inflammatory state toward a reparative program. A deeper mechanistic understanding of this process may provide new therapeutic avenues for refractory wounds, including diabetic ulcers.

#### 4.1.2 Skeletal muscle regeneration

During muscle repair following injury, macrophage polarization is a key determinant of recovery. After muscle damage, macrophages initially adopt an M1-like phenotype to rapidly clear debris and invading bacteria and to produce abundant pro-inflammatory mediators. However, during a critical post-injury window, macrophages must transition from an M1-like to an M2-like phenotype to support tissue repair. In this context, histone H3K18 lactylation accumulates at promoter and enhancer regions of macrophage repair-related genes, thereby activating transcription of genes implicated in regeneration, such as *Lrg1*, *Vegf-a*, and *IL-10 (*56). Expression of these genes promotes fibroblast proliferation, collagen synthesis, and neovascularization, thus establishing a permissive microenvironment for muscle repair and regeneration. Moreover, by regulating these repair-associated transcriptional programs, lactylation not only accelerates local tissue restoration but may also enhance muscle regenerative capacity through modulation of the local immune microenvironment.

#### 4.1.3 Spinal cord injury and functional recovery

Spinal cord injury (SCI) is a devastating neurological disorder, and functional recovery directly affects patients’ quality of life. Following SCI, alterations in the local microenvironment and immune regulation are critical determinants of spinal cord repair. Accumulating evidence indicates that macrophages and microglia play particularly important roles during the post-injury repair process. In microglia, histone H4K12 lactylation (H4K12la) has been shown to promote transcription and enhance expression of *Spp1*, thereby increasing Spp1 levels within the extracellular matrix (57). As an extracellular matrix protein, Spp1 regulates neuronal maturation, optimizes axonal mitochondrial energy metabolism, and modulates inflammatory-cell infiltration by engaging cell-surface receptors. Collectively, these effects help attenuate post-injury inflammation, support neuronal repair and regeneration, and ultimately accelerate functional recovery after SCI. These findings suggest that targeting lactylation may offer new therapeutic opportunities for SCI, particularly by promoting neural regeneration through modulation of microglial activity and immune responses.

### 4.2 Infectious and inflammatory diseases

#### 4.2.1 Acute infection and sepsis

Sepsis is a systemic inflammatory response syndrome triggered by infection and can progress to septic shock and multiple organ dysfunction in severe cases. In sepsis-related settings, methylsulfonylmethane (MSM) has been reported to effectively control methicillin-resistant *Staphylococcus aureus* (MRSA) infection and to prevent MRSA-induced sepsis. Mechanistically, MSM appears to act through a lactate–H3K18 lactylation axis (59): it enhances glycolytic metabolism in peritoneal macrophages, upregulates expression of genes such as *Arg1*, and promotes polarization toward an M2-like phenotype, thereby attenuating systemic inflammation. Conversely, type I interferons suppress lactate production and histone lactylation (60), which in turn induces *iNOS* expression during MRSA infection, facilitating bacterial dissemination and exacerbating systemic inflammatory responses.Clinically, elevated serum lactate levels in patients with sepsis are positively correlated with mortality. At the mechanistic level, lactate accumulation during sepsis promotes lactylation of cold-inducible RNA-binding protein (CIRP) in macrophages (61). Released CIRP subsequently induces apoptosis of pulmonary vascular endothelial cells (PVECs), thereby aggravating sepsis-associated acute lung injury (ALI). In addition, RNA sequencing analyses have identified multiple lactylation-associated genes linked to sepsis and septic shock, including *S100A11* and *CCNA2 (*62, 63). These genes may represent promising intervention targets for sepsis progression and potential biomarkers for septic shock.

#### 4.2.2 Chronic inflammation and barrier-associated diseases

In mice deficient in BCAP (B cell adaptor for phosphoinositide 3-kinase), an upstream regulatory molecule in Toll-like receptor (TLR) signaling, macrophage glycolysis is impaired, leading to reduced lactate production, decreased histone lactylation, and attenuated expression of repair-associated genes (e.g., *Arg1* and *Klf4*). Consequently, macrophages fail to efficiently transition to a reparative phenotype during the late inflammatory phase, which ultimately results in an attenuated response in dextran sulfate sodium (DSS)-induced colitis (64). In addition, TAK-242, a TLR4 inhibitor, has been reported to promote macrophage polarization toward an M2-like phenotype by increasing histone lactylation (65), thereby facilitating mucosal healing and suggesting potential utility in chronic inflammatory diseases. Nevertheless, these findings remain largely limited to experimental and early clinical stages, and further clinical evidence is required to establish safety and efficacy. By contrast, Gegen Qinlian Decoction (GQD), a traditional Chinese medicine frequently used for gastrointestinal disorders, has been shown to inhibit lactate production and lactate dehydrogenase (LDH) activity, thereby suppressing lysine lactylation marks, including H3K18la, H3K23la, H4K8la, and H4K12la. This inhibition limits M1 macrophage polarization and effectively alleviates ulcerative colitis progression (66). Periodontitis (PD) is another chronic inflammatory disease triggered by oral bacteria in dental plaque, such as *Porphyromonas gingivalis* (67). Using immunohistochemistry and Western blotting, researchers have observed that elevated lactylation in periodontitis suppresses transcription of M1-associated polarization factors, while significantly upregulating M2-associated factors, thereby mitigating inflammatory damage in periodontal tissues (68).

These observations should not be interpreted as a simple contradiction in which TLR signaling or lactylation is always either inflammatory or anti-inflammatory. TLR ligation can initiate an early NF-κB-driven inflammatory program, while prolonged glycolytic rewiring may generate lactate and lactylation marks that support later resolution or repair-associated genes. Similarly, *TAK-242, BCAP* deficiency, and GQD appear to influence different nodes of the inflammatory-metabolic axis: receptor-proximal TLR4 signaling, glycolytic competence, and LDH-dependent lactate generation, respectively. Therefore, the functional consequence of lactylation depends on when it occurs during the inflammatory trajectory and on which target genes or proteins become lactylated.

### 4.3 Cardiovascular and metabolic-related diseases

#### 4.3.1 Atherosclerosis and vascular inflammation

Lactylation contributes to the induction of reparative macrophage programs and has been implicated in preventing or attenuating atherosclerosis progression. During atherogenesis, *MCT4* is highly expressed in macrophages. In this setting, *MCT4* inhibition is proposed to limit lactate export, causing intracellular lactate retention, increased histone H3K18 lactylation, and enrichment of this mark at promoter regions of reparative genes. This epigenetic remodeling facilitates macrophage polarization toward an anti-inflammatory or reparative phenotype, thereby exerting protective effects against atherosclerosis (69). This mechanism should be distinguished from conditions in which increased *MCT4* expression in highly glycolytic cells increases extracellular lactate availability and indirectly enhances lactylation in neighboring cells; the direction of the phenotype therefore depends on cell type, lactate gradients, and whether the dominant effect is lactate retention or intercellular lactate shuttling. In addition, exercise has been reported to induce lactylation of methyl-CpG–binding protein 2 (MeCP2) at K271, driving macrophage polarization toward an M2-like phenotype, ameliorating atherosclerosis progression, and reducing mortality in patients with atherosclerotic cardiovascular disease (ASCVD) (3). Notably, lactylation can also exert context-dependent detrimental effects. Lactylation of SRY-related HMG-box transcription factor 10 (Sox10) promotes transdifferentiation of vascular smooth muscle cells (VSMCs) into macrophage-like cells, a process that may drive vascular proliferation and aggravate atherosclerotic complications (70). Accordingly, enhancing MeCP2 K271 lactylation or inhibiting Sox10 lactylation may represent promising therapeutic strategies to improve outcomes and quality of life in patients with ASCVD.

#### 4.3.2 Metabolic cardiomyopathy and inborn errors of metabolism

Diabetic cardiomyopathy, a cardiomyopathy associated with type 2 diabetes mellitus, is characterized by myocardial metabolic derangements, cardiovascular dysfunction, and autonomic neuropathy. During disease progression, elevated free fatty acids induce upregulation of MCT4, leading to extracellular lactate accumulation and promoting histone H4K12 lactylation (71). This lactylation is reported to enhance *HIF-1α* transcription, thereby driving macrophage polarization toward an M1-like phenotype and further aggravating disease progression. Glycogen storage disease type Ib (GSD-Ib) is a rare inherited metabolic disorder caused by deficiency of glucose-6-phosphate translocase (G6PT). G6PT deficiency accelerates glycolysis in macrophages, and the resulting lactate accumulation induces histone H3K18 lactylation (72). Mechanistically, H3K18 lactylation upregulates expression of ALKBH5, reduces N6-methyladenosine (m^6^A) modification of *NLRP3* mRNA, and suppresses activation of the NLRP3 inflammasome in macrophages. This differs mechanistically from macrophage MCT4 inhibition in atherosclerosis: in diabetic cardiomyopathy, increased MCT4 likely reflects enhanced glycolytic lactate export and extracellular lactate accumulation in the injured cardiac niche, whereas in the atherosclerosis model, MCT4 blockade can trap lactate intracellularly. Both scenarios can converge on lactylation, but through distinct lactate-flow configurations.

### 4.4 Tumor

In lung squamous cell carcinoma (LUSC), tumor cells show high expression of solute carrier family 2 member 1 (*SLC2A1*), which encodes glucose transporter 1 (*GLUT1*). This upregulation is closely associated with increased lactylation and promotes polarization of *SPP1*^+^ macrophages toward an M2-like phenotype within the tumor microenvironment, thereby accelerating tumor progression (74). In the gastric cancer microenvironment, elevated H3K18 lactylation enhances *VCAM1* transcription. VCAM1 activation increases *CXCL1* expression via the AKT–mTOR–CXCL signaling axis, which promotes infiltration of gastric cancer–derived mesenchymal stem cells (hGC-MSCs) and M2 macrophages. This remodeling of the microenvironment facilitates gastric cancer cell proliferation, migration, and epithelial–mesenchymal transition (EMT), ultimately contributing to immune evasion and poor prognosis (75). Consistently, higher lactylation scores in gastric cancer are associated with increased M2 macrophage infiltration, worse clinical outcomes, higher tumor grade, and lymph node metastasis (76); in parallel, increasing lactylation scores correlate with stronger proliferative, invasive, and metastatic capacities of gastric cancer cells.Pancreatic ductal adenocarcinoma (PDAC) is often referred to as the “king of cancers”. In the PDAC microenvironment, lactate accumulation induces lactylation of alpha-endosulfine (ENSA) at K63, which activates the ENSA–STAT3–CCL2 axis, promotes recruitment of M2 macrophages, and drives malignant progression (30). In prostate cancer, five lactylation-related genes (LRGs)—*ALDOA*, *DDX39A*, *H2AX*, *KIF2C*, and *RACGAP1*—have been proposed as predictive biomarkers for disease-free survival (DFS) and treatment response (77). Moreover, inhibition of H3K18 lactylation enhances the phagocytic activity of tumor-associated macrophages (TAMs), thereby suppressing tumor growth (86). In bladder cancer, high H3K18 lactylation increases the abundance of *PRKN* in the tumor microenvironment (TME), promotes mitophagy and M2 polarization, and exacerbates immune evasion and tumor progression (87). Similarly, elevated H3K18 lactylation in the TME of epithelial ovarian cancer (OV) induces M2 macrophages to secrete CCL18 (88), which is associated with poorer overall survival (OS) and progression-free survival (PFS) (51). In breast cancer cell lines, histone lactylation has also been reported to promote macrophage polarization toward an M2-like phenotype by activating c-Myc expression and regulating M2-associated gene programs (51).

Glioma is the most common primary brain tumor. Elevated H3K18 lactylation within the tumor microenvironment has been reported to upregulate tumor necrosis factor superfamily member 9 (TNFSF9), thereby promoting macrophage polarization toward an M2-like phenotype and accelerating glioma progression (89). Neuroblastoma (NB) is a malignant tumor of neural crest origin and is most frequently diagnosed in children. In neuroblastoma models, knockdown of hexokinase 3 (*HK3*) in tumor cells reduces lactate production and histone lactylation in the tumor microenvironment (TME), which markedly decreases the proportion of M2 macrophages and consequently suppresses neuroblastoma cell proliferation, invasion, and migration (90).

Taken together, tumor studies indicate that lactylation is not merely a downstream marker of glycolysis but can help stabilize immunosuppressive myeloid circuits. Tumor-cell *SLC2A1*/GLUT1 activity, extracellular lactate accumulation, MCT-mediated lactate exchange, and histone or non-histone lactylation in TAMs converge on transcriptional programs that support angiogenesis, EMT, immune evasion, and therapy resistance. Nevertheless, most tumor evidence remains cancer-type and model specific; therefore, causal claims should be limited to settings in which lactate transport, lactylation enzymes, or defined Kla sites have been directly perturbed and functionally rescued. Future translational studies should combine lactylation profiling with single-cell/spatial mapping of TAM states and T-cell function to define which tumors are most likely to benefit from lactylation-targeted myeloid reprogramming.

### 4.5 Fibrosis-associated diseases

Pulmonary fibrosis is a hallmark pathological feature of interstitial lung disease (ILD) and is typically accompanied by progressive fibrotic remodeling. During fibrogenesis, myofibroblasts generate large amounts of lactate through glycolytic metabolism, and lactate accumulation promotes histone lactylation, thereby further inducing transcription of fibrosis-related genes (79). Consistently, exposure to fine particulate matter (PM2.5) markedly increases lactate dehydrogenase (LDH) activity and lactate levels in mouse lung tissue and bronchoalveolar lavage fluid, while upregulating transcription of key glycolytic enzymes. These metabolic changes elevate histone lactylation at promoters of pro-fibrotic genes in alveolar macrophages (80). As a result, expression of pro-fibrotic genes—including *TGF-β*, *VEGF*, *PDGF*, *Thbs1*, and *Opn*—is increased, accompanied by enhanced secretion of pro-fibrotic cytokines such as TGF-β and VEGFA. Activation of the TGF-β/Smad2/3 and VEGFA/MEK/ERK pathways subsequently induces epithelial–mesenchymal transition (EMT) in MLE-12 alveolar epithelial cells, thereby promoting pulmonary fibrosis progression. Currently, treatment of pulmonary fibrosis still relies largely on lung transplantation; however, donor shortages and immune rejection underscore an urgent need for new therapeutic approaches. Recent evidence suggests that activation of the glucagon-like peptide-1 receptor (GLP-1R)—a target of antidiabetic therapies--can inhibit NLRP3 inflammasome activation and PFKFB3-driven glycolysis while suppressing histone lactylation in lung fibroblasts, thereby exerting anti-fibrotic effects (81). Silicosis is an occupational lung disease caused by long-term inhalation of crystalline silica dust and is characterized by persistent pulmonary inflammation and irreversible fibrosis. Studies indicate that glycolytic metabolic reprogramming in macrophages promotes lactylation, which contributes to crystalline silica–triggered, NLRP3-dependent pyroptosis, thereby driving pulmonary inflammation and silicosis pathogenesis (82). Liver fibrosis is another chronic injury–associated pathological state marked by excessive scar tissue deposition. Salvianolic acid B has been reported to alleviate CCl_4_-induced liver injury and fibrotic progression by reducing histone lactylation in hepatic Kupffer cells (83). Given its broad availability and favorable safety profile, salvianolic acid B has attracted increasing attention as a potential therapeutic agent for liver fibrosis.Collectively, lactylation can contribute to organ structural damage and functional decline by regulating pro-fibrotic gene programs in macrophages. Therefore, therapeutic strategies targeting lactylation may offer a promising avenue for the treatment of fibrosis-associated diseases.

### 4.6 Degenerative diseases of the central nervous system

Alzheimer’s disease (AD) is a common neurodegenerative disorder of the central nervous system and the leading cause of dementia in older adults, with risk increasing markedly with age. Multiple hypotheses have been proposed to explain AD pathogenesis, including the β-amyloid cascade hypothesis, the tau hyperphosphorylation hypothesis, neuroinflammation, oxidative stress, and synaptic dysfunction. In recent years, however, accumulating evidence has suggested that lactylation may also contribute to AD initiation and progression. In 2022, Pan et al. reported elevated histone lactylation in microglia adjacent to β-amyloid plaques in AD mouse models and in brain tissue samples from patients, and this modification was associated with pro-inflammatory microglial activation (84). These observations provide a new perspective on the potential involvement of lactylation in neurodegenerative diseases.

Parkinson’s disease (PD), historically referred to as paralysis agitans, is a common neurodegenerative disorder that primarily affects middle-aged and older individuals. Recent studies suggest that histone H3K9 lactylation (H3K9la) can activate *SLC7A11*, promote microglial activation, and thereby trigger neuroinflammation, ultimately contributing to dopaminergic neuronal damage. Accordingly, inhibition of glycolysis to reduce histone lactylation has been reported to attenuate PD progression (85). These findings highlight a potential therapeutic avenue for PD, namely, slowing neurodegeneration by modulating lactylation levels.

### 4.7 Cross-disease integration: determinants of divergent lactylation outcomes

Across disease contexts, lactylation does not encode a single immunological instruction. Instead, its functional output appears to be determined by at least five interacting variables. First, the timing of lactylation relative to inflammatory activation is critical: delayed lactylation after an early inflammatory burst may promote resolution and repair, whereas sustained lactylation within chronic inflammatory circuits can amplify pathological signaling. Second, lactate source and transport direction matter; autocrine lactate retention, paracrine tumor-derived lactate, and systemic metabolic lactate are not equivalent. Third, the targeted substrate and residue determine function, because H3K18la/H4K12la-driven transcriptional programs, non-histone lactylation of HMGB1, PKM2, ENSA, MeCP2, or Sox10, and reader-dependent chromatin interpretation can have different consequences. Fourth, macrophage ontogeny and tissue identity influence chromatin accessibility and therefore which genes are permissive to lactylation. Fifth, disease-specific microenvironments, including hypoxia, TLR ligands, cytokines, fatty acids, amyloid plaques, silica particles, or tumor metabolites, determine whether lactylation stabilizes reparative, immunosuppressive, pro-fibrotic, or pro-inflammatory macrophage states. These considerations help reconcile apparently conflicting observations in wound repair, cancer, fibrosis, Alzheimer’s disease, and Parkinson’s disease.

## 5 Lactylation-mediated regulation of macrophage polarization

### 5.1 Lactylation upregulates M2 macrophage signature genes

Histone lactylation can remodel chromatin architecture and accessibility, thereby modulating transcription factor occupancy at gene promoters and directly upregulating M2 macrophage signature genes to promote polarization toward an M2-like phenotype (91). For example, following myocardial infarction, H3K18 lactylation (H3K18la) is elevated in macrophages both within the infarcted myocardium and in distal peripheral compartments, initiating expression of reparative genes such as *Lrg1*, *Vegf-a*, and *IL-10 (*58). In the tumor microenvironment, lactate produced by cancer cells similarly increases H3K18la in macrophages and enhances transcription of M2-associated genes (92). Consistently, in tumor-associated macrophages from B16F10 melanoma and LLC1 lung tumors, histone lactylation levels are positively correlated with Arg1 expression (1). Moreover, upregulation of SRSF10 (a member of the serine/arginine-rich splicing factor family) in hepatocellular carcinoma has been reported to promote lactate production and elevate H3K18la, thereby activating transcription of M2 signature genes (93). In fibrotic settings, lactylation also drives polarization toward a pro-fibrotic program. During pulmonary fibrosis, lactylation accumulates at promoters of multiple pro-fibrotic genes (e.g., *Arg-1*, *PDGFA*, *THBS1*, and *VEGFA*), promoting their expression in alveolar macrophages and biasing macrophages toward a pro-fibrotic phenotype (79). Similar mechanisms have been described in periodontitis (68), atherosclerosis (69), and sepsis (59). Furthermore, non-histone lactylation can exert analogous regulatory effects, as exemplified by the protective role of MeCP2 K271 lactylation in atherosclerosis (3) and the immunomodulatory function of HMGB1 lactylation in liver ischemia–reperfusion (I/R) injury (94). Collectively, these studies support the concept that targeting lactylation to reprogram macrophage polarization may represent a promising strategy for disease intervention.

### 5.2 Lactylation-driven reprogramming of transcription factors and signaling networks

A complex network of transcription factors and signaling pathways orchestrates macrophage polarization. Lactylation can fine-tune macrophage polarization by modulating key transcription factors and their downstream signaling cascades. In type 2 diabetic cardiomyopathy (71) and Alzheimer’s disease (73), elevated histone H4K12 lactylation (H4K12la) has been observed in macrophages and is associated with activation of hypoxia-inducible factor-1α (HIF-1α), skewing macrophages toward an M1-like phenotype and exacerbating inflammatory responses. In colorectal cancer, increased H3K18 lactylation (H3K18la) has been reported to enhance expression of nuclear factor-κB (NF-κB), which subsequently activates STAT3 signaling, promotes M2-like polarization, and facilitates tumor progression (95). In breast cancer patients, histone lactylation at the promoter of *c-Myc* is significantly higher than that in healthy individuals and is closely associated with macrophage polarization toward an M2-like phenotype (51). Moreover, lactylation of retinoic acid–inducible gene 1 (RIG-1) suppresses NF-κB signaling, reduces the abundance of M1 macrophages, and promotes colorectal cancer liver metastasis (96). Collectively, these studies indicate that lactylation can regulate macrophage polarization through coordinated control of transcription factor expression and signaling pathway activation, thereby shaping immune responses and tumor progression.

### 5.3 Lactylation regulates metabolism-related gene expression

Lactylation can reshape the transcriptional landscape of metabolism-related genes by remodeling chromatin accessibility and/or directly modulating the activity of transcriptional complexes. In doing so, lactylation influences the direction of macrophage metabolic reprogramming and thereby biases polarization outcomes. In 2020, shortly after the concept of lactylation was introduced, Gaffney D. O. et al. proposed that lactylation may negatively regulate glycolysis by modifying glycolytic enzymes and suppressing their activity. This reduction in enzymatic activity would decrease glycolytic metabolite levels and favor a transition toward a reparative macrophage phenotype (97). Subsequent studies have provided support for this model, including evidence that PKM2 lactylation at K62 participates in glycolytic regulation (29). In addition, lactate generated during mitochondrial fragmentation has been reported to induce histone lactylation and increase expression of arginase 1 (*Arg1*); as a key metabolic enzyme characteristic of M2 macrophages, Arg1 promotes polarization toward an M2-like phenotype (98). Notably, alternative patterns have also been described. In patients with Alzheimer’s disease, elevated H4K12 lactylation (H4K12la) has been linked to activation of a glycolysis–H4K12la–PKM2 positive feedback loop, driving microglia toward a pro-inflammatory phenotype (73). Together, these observations suggest that the effects of lactylation on metabolic gene programs and macrophage polarization are context dependent and may operate through distinct mechanisms across different disease settings, thereby differentially shaping disease progression.

Metabolic-epigenetic feedback circuits may therefore represent a general organizational principle rather than a phenomenon restricted to Alzheimer’s disease. In the glycolysis-H4K12la-PKM2 loop described in AD, enhanced glycolysis increases lactate availability, H4K12la facilitates expression of glycolytic regulators, and PKM2 further sustains inflammatory metabolic adaptation (73). Analogous, although not necessarily identical, circuits may operate in tumor microenvironments: tumor-cell glycolysis supplies extracellular lactate, macrophage lactate uptake or retention enhances histone lactylation, and lactylation-dependent expression of genes such as *VCAM1*, *CXCL1*, *CCL2*, *Arg1*, or *SPP1*-associated programs can recruit or maintain immunosuppressive macrophage states (30, 75, 78, 92).

However, the strength of evidence differs among settings. The AD circuit has relatively direct mechanistic support for a positive feedback loop, whereas many TME studies currently show pathway associations, ChIP enrichment, lactylation scores, or pharmacological perturbation rather than residue-specific causal closure. Future work should test whether disrupting individual nodes—LDH/MCT-dependent lactate supply, p300/CBP or KAT-mediated writing, HDAC/SIRT-mediated erasure, or reader-mediated chromatin interpretation—can collapse these circuits and durably reprogram TAMs without suppressing antitumor T-cell immunity.

### 5.4 Crosstalk between histone lactylation and other epigenetic modifications

Histone lactylation and other epigenetic modifications can coordinately regulate macrophage activation through intricate crosstalk, producing synergistic or antagonistic effects. This crosstalk may occur at several levels. First, lactylation can compete with other lysine acylations, especially acetylation, for the same or adjacent lysine residues and for shared enzymes such as p300/CBP, HBO1/KAT7, KAT8/MOF, and class I HDACs (1, 26, 33, 34). Second, lactylation can cooperate with permissive histone marks to increase chromatin accessibility or transcription-factor recruitment, while antagonizing repressive marks depending on the locus. Third, histone lactylation may regulate RNA-modification machinery; for example, lactylation-driven expression of *ALKBH5* or *METTL3*-associated pathways links lactate metabolism to m6A dynamics and inflammatory gene expression (72). Fourth, non-histone lactylation can intersect with acetylation, phosphorylation, ubiquitination, and autophagy-related modifications by changing the stability, localization, or activity of signaling proteins such as HMGB1, PKM2, SQSTM1, ENSA, MeCP2, and Sox10 (2, 3, 29, 30, 70).

Such multilayered crosstalk between lactylation and diverse epigenetic modifications has also been validated in biological models of macrophage phenotypic switching. In a study examining macrophage polarization under cold stress, cold exposure increased histone acetylation at the promoter of NF-κB while simultaneously elevating histone lactylation at the promoter of STAT6. Together, these modifications contributed to macrophage adaptation to cold stress and promoted bidirectional activation toward both inflammatory and repair-associated programs (99). This example illustrates why lactylation effects are context-dependent: the same lactate-derived mark may reinforce or counterbalance other epigenetic signals depending on the local chromatin state, target locus, and inflammatory timing. A more complete understanding of these interactions will require multi-omics approaches that integrate lactylome profiling, chromatin accessibility, histone-mark mapping, transcriptomics, and single-cell macrophage-state analysis.

## 6 Therapeutic targeting of lactylation to modulate macrophage polarization

Lactylation appears to preferentially drive macrophage polarization toward an M2-like phenotype by reshaping epigenetic programs and reconfiguring intracellular signaling pathways. Notably, its biological effects are disease-context dependent: lactylation may promote tissue repair and anti-inflammatory responses, yet under certain circumstances it can also facilitate disease progression. Accordingly, strategies that increase lactylation—such as local administration of exogenous lactate, enhancement of glycolytic flux, and inhibition of HDAC activity—may promote M2 polarization, suppress excessive inflammation, and support angiogenesis and tissue remodeling, highlighting potential therapeutic value in reparative and inflammatory disorders.Glycolytic activity can be augmented by increasing the activity of key rate-limiting enzymes, including hexokinase (HK), phosphofructokinase-1 (PFK-1), pyruvate kinase M2 (PKM2), and lactate dehydrogenase (LDH). Broad-spectrum HDAC inhibitors that have been identified include trichostatin A (TSA) (100) and vorinostat (SAHA) (101); notably, SAHA has been approved by the U.S. Food and Drug Administration (FDA) and is used clinically for the treatment of cutaneous T-cell lymphoma. HDAC3 is considered a particularly important delactylase, and the development of selective HDAC3 inhibitors has therefore become an active area of investigation. In disease contexts such as cancer, fibrosis, and central nervous system degenerative disorders, strategies aimed at lowering lactylation—including inhibition of lactate dehydrogenase (LDH) to reduce lactate production, blockade of lactate transport using MCT1/4 inhibitors, and enhancement of HDAC activity—have been reported to decrease macrophage lactylation, reverse macrophage-mediated immunosuppression, slow disease progression, and improve prognosis. To date, multiple LDH inhibitors have been identified, including ammonium oxalate (102), quinoline-3-sulfonamides (103), 2-thioxo-6-oxo-1,6-dihydropyrimidines (104), 2-amino-5-arylpyrazines (105), and GF (106). Although these compounds still face challenges such as limited efficacy and off-target effects, ongoing and future clinical evaluation of LDH inhibition may provide new therapeutic opportunities for refractory diseases. In addition, accumulating evidence indicates that monocarboxylate transporter (MCT) inhibitors—such as 7-aminocoumarin derivatives (107), the small-molecule MCT1 inhibitor AZD3965 (108), the arylacetic acid nonsteroidal anti-inflammatory drug diclofenac (109), ciproxifan (110), and lonidamine (LND)—can inhibit MCT function and disrupt the associated glycoprotein complex CD147/BASIGIN (BSG), thereby representing a potential anticancer strategy (111). At present, selective activators of HDAC3 remain scarce, representing an important direction for future research. Moreover, lactylation-targeted strategies may be combined with PD-1 blockade, chemotherapy, radiotherapy, and other treatment modalities, potentially offering synergistic benefits in cancer therapy. Recent studies have also reported that lactylation levels correlate with disease progression across multiple disorders, including hepatocellular carcinoma (112), prostate cancer (77), diffuse large B-cell lymphoma (113), nasopharyngeal carcinoma (114), and sepsis (62). Future work should explore whether precise control of lactylation—potentially through direct gene knockout or transcriptional upregulation of specific genes—can steer macrophage polarization and thereby further mitigate disease progression.

### 6.1 Therapeutic window and cellular selectivity

A major translational challenge is that lactate metabolism is not macrophage-specific. Effector T cells, NK cells, endothelial cells, fibroblasts, and tumor cells all use glycolysis and lactate transport under activation or stress. Systemic inhibition of LDH or MCTs may therefore reduce macrophage lactylation but could also alter adaptive immune effector functions, including the glycolytic programs required for T-cell proliferation, cytokine production, and cytotoxicity (7, 115). Conversely, in selected tumor contexts, reducing tumor-derived lactate may relieve lactate-mediated suppression of T and NK cells and improve checkpoint blockade responses (6–8, 115). The therapeutic window will depend on whether the dominant effect is inhibition of immunosuppressive myeloid lactylation, tumor metabolic restriction, or unintended suppression of lymphocyte metabolism.

Several strategies may improve cellular selectivity. First, local or tissue-targeted delivery, including intratumoral administration, inhaled delivery for lung disease, or macrophage/TAM-targeted nanoparticles, could reduce systemic exposure. Second, treatment schedules could be optimized so that lactate-pathway inhibition is transient or synchronized with immunotherapy, allowing recovery of effector lymphocyte metabolism. Third, therapies could target downstream lactylation machinery, reader interactions, or disease-specific substrates rather than globally depleting lactate. Fourth, patient selection using lactate levels, lactylation signatures, single-cell myeloid states, and T-cell functional readouts may identify settings in which myeloid reprogramming is likely to outweigh systemic metabolic toxicity. Finally, combination strategies should be evaluated with immune-monitoring endpoints, including CD8+ T-cell glycolytic competence, cytokine secretion, and cytotoxicity, rather than tumor or macrophage endpoints alone.

In summary, therapeutically targeting lactylation to modulate macrophage polarization represents an emerging strategy for reshaping the immune microenvironment. This approach provides new therapeutic opportunities across a broad spectrum of diseases and may be particularly promising for cancer, inflammatory disorders, fibrosis, and degenerative conditions.

## 7 Conclusion

As an emerging epigenetic mechanism, lactylation has important biological implications in regulating macrophage polarization and function. Accumulating evidence indicates that lactylation not only shapes macrophage polarization by remodeling gene-expression programs and metabolic reprogramming, but also contributes to the initiation and progression of diverse diseases. Across pathological contexts—including tissue repair, immune evasion, tumor progression, chronic inflammation, and fibrosis—lactylation can influence disease outcomes by modulating the M1/M2 polarization balance and thereby promoting or restraining specific immune responses.

Of note, the divergent biological outcomes of lactylation arise from multiple quantitative and qualitative determinants, and cannot be explained merely by global lactylation levels. These include lactate concentration and exposure duration, intracellular versus extracellular lactate flux, tissue oxygenation, inflammatory timing, macrophage ontogeny, available writer/eraser enzymes, reader-protein expression, chromatin accessibility, and the identity of lactylated substrates. Thus, a modest, transient lactylation signal after acute inflammation may promote repair, whereas sustained lactylation in tumors, fibrotic tissue, or neurodegenerative lesions may stabilize immunosuppressive, pro-fibrotic, or pro-inflammatory circuits. Future studies should therefore move beyond binary M1/M2 readouts and incorporate single-cell and spatial analyses to define how lactylation remodels macrophage states *in vivo*.

From a translational perspective, manipulating macrophage polarization through lactylation offers promising therapeutic targets. Promoting macrophage polarization toward an M2-like phenotype may alleviate inflammation and support tissue repair and angiogenesis, suggesting broad potential for treating reparative conditions and chronic inflammatory diseases. By contrast, in immune-evasive diseases such as cancer, inhibiting lactylation may help restore immune surveillance and strengthen anti-tumor responses. Moreover, lactylation-targeted approaches may be integrated with established therapies—including PD-1 blockade, chemotherapy, and radiotherapy—to potentially expand therapeutic benefit in cancer and other disorders. Despite increasing evidence supporting a critical role for lactylation in immune regulation, its precise mechanisms remain incompletely defined, particularly with respect to how lactylation can be modulated in a controlled manner to optimize macrophage function. Future studies should prioritize elucidation of lactylation “writers” and “erasers” and delineation of lactylation crosstalk with other epigenetic modifications, which will be essential for advancing both mechanistic understanding and clinical translation.

In summary, lactylation-mediated regulation of macrophage polarization not only provides new insights into the immune regulatory network but also highlights promising therapeutic avenues for immunomodulatory treatment of related diseases.
